# Neuromuscular and biomechanical functions subserving finger dexterity in musicians

**DOI:** 10.1038/s41598-019-48718-9

**Published:** 2019-08-21

**Authors:** Yudai Kimoto, Takanori Oku, Shinichi Furuya

**Affiliations:** 1 Sophia University, Tokyo, Japan; 2 Sony Computer Science Laboratories Inc. (SONY CSL), Tokyo, Japan

**Keywords:** Motor cortex, Neurophysiology, Human behaviour

## Abstract

Exceptional finger dexterity enables skillful motor actions such as those required for musical performance. However, it has been not known whether and in what manner neuromuscular or biomechanical features of the fingers subserve the dexterity. We aimed to identify the features firstly differentiating the finger dexterity between trained and untrained individuals and secondly accounting for the individual differences in the dexterity across trained individuals. To this aim, two studies were conducted. The first study compared the finger dexterity and several neuromuscular and biomechanical characteristics of the fingers between pianists and non-musicians. As a measure of the dexterity, we used the maximum rate of repetitive finger movements. The results showed no differences in any biomechanical constraints of the fingers between the two groups (i.e. anatomical connectivity between the fingers and range of motion). However, the pianists exhibited faster finger movements and more independent control of movements between the fingers. These observations indicate expertise-dependent enhancement of the finger dexterity and reduction of neuromuscular constraints on movement independence between the fingers. The second study assessed individual differences in the finger dexterity between trained pianists. A penalized regression determined an association of the maximum movement speed of the fingers with both muscular strength and biomechanical characteristics of the fingers, but not with neuromuscular constraints of the fingers. None of these features covaried with measures of early and deliberate piano practice. These findings indicate that distinct biological factors of finger motor dexterity differentiate between the effects of piano practicing and individual differences across skilled pianists.

## Introduction

Ubiquitous features of the hand provide dexterity that enables skillful manipulation of various tools, an ability that is necessary for medical procedures, manufacturing, and performing arts such as musical performance. Manual dexterity represents the ability consisting of multifaceted elements of motor skills, such as speed, spatiotemporal accuracy, and complexity of finger movements. A number of studies have attempted to identify neural and biomechanical attributes that play roles in producing dexterous finger movements^1^, which includes neuromuscular functions^2^, inhibitory and excitatory functions of motor cortical neurons^3–5^, and the anatomical architecture of the hand^6–8^. Most of these biological features can be adapted to some extent through training^9^. However, this sometimes results in maladaptive changes in the sensorimotor system and causes movement disorders such as focal dystonia^10,11^. In addition, many movement disorders, such as stroke, cerebellar dysfunctions, and carpal tunnel syndrome, degrades manual dexterity, which severely impairs the quality of life^12,13^. For optimizing training and rehabilitation for the finger dexterity, it is inevitable to shed light on neural and biomechanical mechanisms behind it.

Independent motor control between the fingers plays a key role in dexterity of the hand^14–17^. The inter-finger independence allows for the simultaneous production of multiple motor actions by the individual fingers, such as moving one finger while doing the preparatory motion of another finger during a sequential finger movement. This motor skill thus provides a basis of variation of movement repertories^18^. The independent finger motor control is determined by neuromuscular and biomechanical constraints on the fingers, both of which are independent with together^19^. The neuromuscular constraints can originate from the synchronous firing of motor neurons innervating into multiple compartments of the same finger muscle^20–22^ and the shared representation of individual fingers in the motor cortex^23,24^. These functional and anatomical linkages between multiple fingers in the nervous system make it difficult to move one single finger independently from the others. Surround inhibition is a mechanism that inhibits the excitability of motor cortical neurons innervating into a finger adjacent to a finger in use^25^; surround inhibition is therefore likely associated with the production of the individuated finger movements^26^. The biomechanical constraints on the finger dexterity are the anatomical linkages between the tendons and muscles of the hand and forearm^6,27^. Due to such linkages and their viscoelastic characteristics, production of muscular force at one finger accompanies force production at the adjacent fingers^17^. Despite these constraints, highly-skilled movements such as playing the piano are typically characterized by exceptionally independent movements between the fingers, which outperforms those of untrained individuals^18^. One unanswered question is whether such independent motor control is associated with adaptive changes in the neuromuscular or biomechanical constraints of the fingers. Neuroplastic adaptations in the sensorimotor system responsible for skillful finger movements have been observed in musicians^9,28,29^. However, several studies reported reduced surround inhibition of the finger muscles in musicians compared with non-musicians^26,30^, which may predict an increase in neuromuscular constraints. As for the biomechanical constraints, little has been known about the anatomical characteristics of the fingers of trained musicians. However, a previous study reported greater finger mobility in pianists than non-musicians, which raises a possibility that some anatomical changes specific to the former individuals reduce biomechanical constraints on individual fingers^31^.

Another important factor of the manual dexterity is the muscular strength. It is possible that stronger force production of a finger muscle results in faster unidirectional movements of the finger. We demonstrated that an individual difference in the maximum rate of repetitive flexion-extension motions of the upper-limb across expert pianists was associated positively with strength of the elbow extensor muscle^32^. The muscular strength can be also associated with independent motor control between the fingers, since the individuated finger movements can necessitate voluntary muscular force production for suppressing unwanted finger movements originating from the inter-digit connection.

The primary purpose of the present study is to define the expertise-dependent characteristics of the hand neuromuscular and biomechanical functions in pianists capable of highly individuated finger movements by comparing with musically-untrained individuals (i.e. study 1). However, even among trained pianists, there are large inter-individual differences in motor dexterity^33,34^. In addition to deliberate practice^35^, recent studies demonstrated that innate biological features underlie individual differences in musicians’ motor expertise^36–38^. This raises the possibility that even neuromuscular and biomechanical features that have no differences between pianists and non-musicians can explain an individual difference in the finger motor dexterity across trained pianists. The secondary purpose of the study is therefore to identify the neuromuscular and biomechanical features of the hand in association with an individual difference in the finger motor dexterity across trained pianists (i.e. study 2). Among multifaceted features of the finger dexterity, the present study focused on agility of the finger movements. A number of evidences of neuroplastic adaptation of the motor system through musical training let us postulate reduced neuromuscular constraints of the fingers in the pianists as compared to the non-musicians, which subserves faster finger movements in the pianists. Based on our recent observation of reorganization of muscular coordination between the fingers in association with a training-induced increase in the speed of the sequential finger movements^39^, we also postulated smaller neuromuscular constraints of the fingers in pianists who can move fingers faster. In addition, we hypothesized a relation of the individual difference in the finger movement speed to the finger muscular strength.

## Material and Methods

### Participants

The present study consisted of two studies. In study 1, 10 right-handed pianists (5 female) and 10 age-matched right-handed non-musicians (5 female) took part in the experiments (Table 1). In study 2, 14 right-handed pianists (21.1 ± 1.3 years, 9 female) participated. All pianists majored in piano playing and underwent formal musical education at music conservatories, whereas the non-musicians had no experience of playing any musical instruments. We also asked the pianists their history of piano practicing (i.e., the age at which piano practicing was initiated and their average practice time at each age) in each pianist. Based on the average practice time at each age, the total practice time throughout a life was calculated. In accord with the Declaration of Helsinki, the experimental procedures were explained to all participants. Informed consent was obtained from all participants prior to participation in the experiment. The experimental protocol was approved by the ethics committee at Sophia University, and all methods were performed in accordance with the relevant guidelines and regulations.

**Table 1:** Information on participants of the study 1.

	Pianists	Non-musicians	Welch two sample t-test
Number of participants	10	10	
Age (years)	21.0 ± 1.4	22.2 ± 2.0	t(16.248) = −1.555, p = 0.139
Handedness (laterality quotients)	79.7 ± 33.8	88.3 ± 17.3	t(13.946) = -0.705, p = 0.493
Training start age (years)	4.3 ± 1.3	N/A	
Total training time (hours)	15095.5 ± 4573.8	N/A	

Handedness was assessed by the Edinburgh handedness test.

### Experimental tasks

The experiment consisted of assessments of various functions of the right hand. This included the individuated movements, maximum isometric force production, maximum rate of repetitive tapping movements (maximum finger tapping rate), and range of motion of each of the individual fingers. Here, the individual finger movements were decomposed into neuromuscular and biomechanical constraints on the fingers, which are indexed as ACI and PCI, respectively (details follow at the Data Analysis section). These assessed functions were categorized into four groups; (1) manual dexterity: the maximum finger tapping rate, (2) neuromuscular constraints on the fingers: ACI, (3) biomechanical constraints on the fingers: PCI and range of motion, and (4) muscular strength: maximum isometric force production. Although it is possible that the muscular strength is associated with neuromuscular and biomechanical constraints, we separately dealt with them and tested their relationship in the present analysis. During the assessments of study 1, the trunk was in an upright position with minimal movement while keeping the shoulder flexed to 20 degree and the elbow flexed to 90 degree. Each participant was asked not to move the upper limb joints.

#### Maximum finger tapping rate

To assess the manual dexterity, each participant repetitively tapped the force sensor as fast as possible for 10 seconds with each of the fingers in each of different directions (i.e. flexion, extension, leftward, rightward). The forearm was pronated (i.e. palm down), and the fingers were at the neutral position. The location of the sensors was adjusted so that the distal phalanx could exert the force to the sensor. The wrist was immobilized using Velcro tape so that the wrist movements could not affect the finger motions. The total number of the taps per a second was defined as the maximum tapping rate by each finger for each direction. As a measure of dexterity, we used the fastest tapping with each of the fingers in different directions, firstly because the non-musicians could not perform a complex sequences of finger movements, and secondly because this isolated motor action is not largely confounded by complex cognitive processes (e.g. chunking, co-articulation).

#### Assessment of the individuated finger movements

We investigated active and passive individuated finger movements in each participant. Each participant was seated and put his/her forearm on a table with the shoulder in 20 degree of flexion, and with the elbow flexed to 90 degree. The forearm was pronated and the wrist was in a neutral position. For the active motion condition, each participant voluntarily alternately flexed and extended each of his or her fingers at a paced tempo, with a metronome providing a tempo of 184 beat per minute (BPM) (i.e. either flexion or extension per a beat). This pace is equivalent to one used in a previous study (i.e. 3 Hz = 180 BPM at the externally-paced motion^40^). The instructed range of motion (i.e. joint excursion) was approximately 60 degrees of the metacarpophalangeal (MCP) joint. The participants were instructed to move without looking at their hand and to keep the remaining fingers unmoved as much as possible^27^. Five trials were performed for each of the index, middle, ring, and little fingers. For the passive motion condition, each participant wore a custom-made hand exoskeleton that generated force to the proximal-phalange of each finger so as to flex and extend the finger (exiii, Inc.). The exoskeleton moved each finger at the same pace as the active condition (i.e. 184 BPM) with angular resolution <1.0°. Note that this exoskeleton was customized to the study but not to each participant. In both the active and passive motion conditions, we recorded dynamic changes in the finger joint angles by using sensors embedded in a right-handed custom-made glove (CyberGlove III, CyberGlove Systems Inc.). We recorded the motions at 15 degrees of freedom with angular resolution <0.5°, at 8.3 msec intervals (i.e., sampling frequency = 120 Hz). The measured angles were the metacarpophalangeal (MCP) and proximal phalangeal (PIP) joint angles of the four fingers.

#### Range of motion

The maximum range of motion for the leftward and rightward directions (i.e. lateral motion) of each of the fingers was evaluated in each of active and passive conditions. In the active condition, each participant was asked to move each finger to the left and right maximally while keeping the hand open as much as possible. In the passive condition, the experimenter moved a finger of each participant to the left and right while keeping the hand open as much as possible. In both conditions, the forearm was pronated with the palm facing down. The postural muscular contraction was asked to be minimized. The difference of the angles between the leftward and rightward motions was defined as the range of motion for each finger.

#### Maximum force production test

Each participant was asked to exert the maximal force isometrically in each of four different directions (i.e. flexion, extension, leftward, rightward) over three seconds with each of the four fingers. The forearm was pronated (i.e. palm down), and the fingers were at the neutral position. The location of the sensors was adjusted so that the distal phalanx could exert the force to the sensor. We recorded the exerted force using a custom-made force sensor system (Leading-Edge Research and Development Accelerator, Inc.) by 1 kHz^34^. The resolution and maximum measurable force of the sensor were 0.05 and 49 N, respectively. During the isometric force production by the finger, the participants were instructed to keep the remaining four digits immobilized voluntarily. The wrist was immobilized using Velcro tape so that the wrist movements could not affect the force production. The peak value of the force production was defined as the maximum force.

### Data analysis

Using the finger kinematic data derived from the data-glove during the individuated finger movements, we computed the individuation index (II), active contribution index (ACI), and passive contribution index (PCI), based on the methods established in a previous study^27^. For each trial, the average angular excursion at each of the MCP and PIP joints of each finger was computed. The II is a measure of the independent movement of a single finger that was instructed to be moved without moving the other fingers. The II was defined as 1 minus the relative joint excursions in the fingers that were instructed to remain immobile (i.e. noninstructed fingers), as follows:

$${\rm{II}}=1-({E}_{ni}/{E}_{i})$$

where E_ni_ is the average excursion of both the MCP and PIP joints of the noninstructed fingers, and E_i_ is the average excursion of both the MCP and PIP joints of the instructed finger. The II will be akin to 1 for an ideally individuated movement where the instructed finger moved without any movement of the non-instructed fingers. The closer the II is to 0, the more noninstructed finger movement occurred together with the instructed finger movement.

To assess the effects of biomechanical coupling of the fingers on finger independence, a PCI was computed for each instructed finger according to the joint excursion value in the passive motion condition. Here, the PCI was defined as the average angular excursion of the joints of the noninstructed fingers expressed as a fraction of the average excursion of the MCP and PIP joints of the instructed finger, as follows:

$${\rm{PCI}}={\rm{passive}}\,{{\rm{E}}}_{{\rm{ni}}}/\text{passive}\,{{\rm{E}}}_{{\rm{i}}}$$

The PCI will be close to 0 with smaller motions of the noninstructed fingers and will increase with larger motions of the noninstructed fingers in the passive condition.

To quantify impacts of neuromuscular control on finger independence, an ACI was computed for each of the fingers based on joint excursion values derived from the passive and active conditions. The ACI was defined as the relative excursion of the MCP and PIP joints of the noninstructed fingers in the active motion condition minus the PCI, as follows:

$${\rm{ACI}}=({{\rm{activeE}}}_{{\rm{ni}}}{/\text{active}\text{E}}_{{\rm{i}}})-{\rm{PCI}}$$

The ACI will be close to 0 with smaller motions of the noninstructed fingers or if they moved the same amount in both the passive and active motion conditions. The ACI will increase with an increase in the noninstructed finger movement in the active motion condition beyond that seen in the passive motion condition.

#### Statistics

The present study evaluated the following variables; II, PCI, ACI, maximum force, maximum finger tapping rate and its coefficient of variation (CV), and range of motion of each of the index, middle, ring, and little fingers.

In study 1, a group-wise comparison was performed statistically. For each of the II, PCI, and ACI, two-way mixed-design analysis of variances (ANOVA) with independent variables using finger (4 levels: index, middle, ring, and little fingers) and group (2 levels: pianists and non-musicians) were performed. A Kolmogorov-Smirnov test confirmed a normal distribution of each of all variables evaluated for each group (p > 0.05). For each of the maximum force, maximum finger tapping rate and its CV of all movement directions (flexion, extension, leftward, and rightward), multiple analysis of variance (MANOVA) using Pillai’s trace statistics were performed. MANOVA was also performed for the range of motion in the active and passive conditions, which used finger and group as independent variables. Post-hoc tests with correction for multiple comparisons^41^ were performed in the case of significant results of ANOVA and MANOVA. These statistical analyses were performed with R statistical software (“manova” function for MANOVA and “ezPerm” function for ANOVA). Among these behavioral measures, a primary measure of the finger dexterity was the maximum finger tapping rate (i.e. agility of the finger movements), whereas a secondary measure was its CV in order to evaluate a speed-accuracy tradeoff.

#### Penalized elastic net regression

In study 2, we tested whether the inter-individual differences in the measure of the dexterity (defined as the maximum finger tapping rate) of the individual fingers were associated with those in the measures of neuromuscular (i.e. ACI) and biomechanical constraints on the fingers (i.e. PCI, range of motion) and the finger muscular strength across pianists. We performed penalized multiple regression analyses with L1 and L2 norm regularization in a linearly combined fashion, so called elastic net regression^42^. We used this penalized regression method firstly because it works independently of multicollinearity between the variables and secondly because the analysis is robust even with a limited number of samples. Also, this regression is not a conventional statistical test but machine learning, and thus neither correction for tests with multiple variables and data distribution needs to be concerned. The analysis was performed with R (“glmnet” function). Both the λ parameter that determines the overall intensity of regularization and the α parameter that specifies a ratio between the L1 and L2 regularization were optimized through an iteration of ten-fold cross-validation of the regularized regression model so as to minimize the least-squared error of the model. The independent variables in the regression model included both age at which piano practicing was commenced and the total amount of life-long piano practicing of the pianists, which were derived from a questionnaire (Table 1). The dependent variable used in the regression model was the maximum tapping rate by a finger and a direction with a significant groupwise difference identified by study 1.

## Results

### Study 1: Comparison between pianists and non-musicians

**Figure 1:**
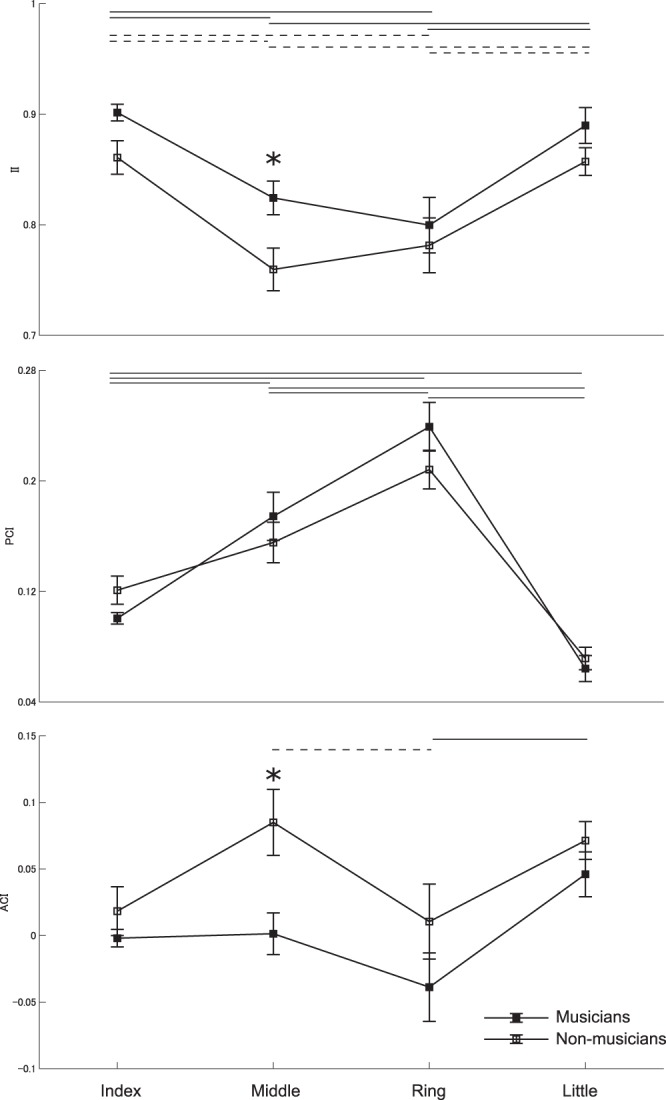
Group means of the individuation index (II), passive contribution index (PCI), and active contribution index (ACI) at the index (I), middle (M), ring (R), and little (L) fingers in the pianists (filled square) and non-musicians (open square). A solid and dotted horizontal line indicates a significant difference between the fingers and between the pianists and non-musicians, respectively (p < 0.05). * significant group difference (p < 0.05). The higher value of the II and ACI indicates higher movement individuation and neuromuscular constraints on the fingers, respectively. An error bar indicates one SEM.

Figure 1 illustrated the group means of the II, PCI, and ACI of the index, middle, ring, and little fingers in the pianists and non-musicians. Two-way mixed-design ANOVAs yielded no interaction effect between group and finger (F(3, 54) = 0.90 and 1.47 and p = 0.448 and 0.232 for the II and ACI, respectively) but significant main effects of both group (F(1, 18) = 4.71 and 5.38 and p = 0.044 and 0.032 for the II and ACI, respectively) and finger (F(3, 54) = 23.64 and 7.71 and p = 6.649 × 10^−10^ and 2.223 × 10^−4^ for the II and ACI, respectively). Post-hoc tests identified a significant group difference in the II and ACI only for the middle finger, showing higher movement independence and smaller neuromuscular constraint for this finger for the pianists than the non-musicians. For the PCI, neither an interaction effect (F(3, 54) = 1.59 and p = 0.201) nor a main effect of group (F(1, 18) = 0.484 and p = 0.496) was significant, which confirmed no group difference for any of the fingers.

**Figure 2:**
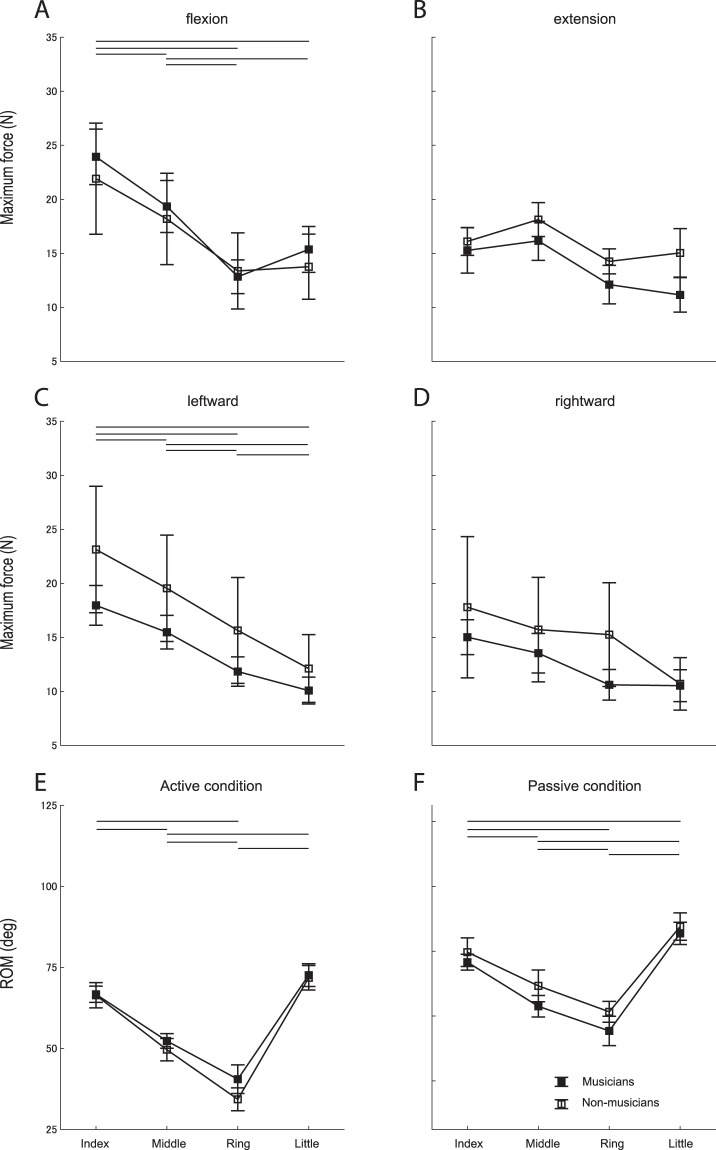
(**A**–**D**) Group means of the maximum force exerted by the index (I), middle (M), ring (R), and little (L) fingers in each of the flexion, extension, leftward, and rightward directions in the pianists (filled square) and non-musicians (open square). The leftward and rightward directions indicate when the hand is pronated so that the palm faces down. (**E**,**F**) Group means of the range of lateral motion of each finger at the active (**E**) and passive (**F**) conditions in the pianists (filled square) and non-musicians (open square). None of these variables showed a significant group difference. A solid horizontal line indicates a significant difference between the fingers in all participants pooled (p < 0.05).

Figure 2 shows the group means of the maximum force exerted in four different directions and the range of lateral motion of the fingers at the active and passive conditions in the pianists and non-musicians. For the maximum force, MANOVA showed that neither interaction effect between group and finger (F(4, 16) = 0.52, p = 0.937) nor group effect (F(1, 4) = 2.33, p = 0.062) was significant. For the range of motion, although MANOVA revealed a significant group effect (F(1, 2) = 5.32, p = 0.007), ANOVAs did not yield any significant group effect at the active (F(1, 2) = 1.03, p = 0.310) and passive (F(1, 2) = 2.50, p = 0.120) conditions. These findings confirmed no group difference in the finger muscular force and range of motion for any of the fingers between the pianists and non-musicians. MANOVA also showed a significant finger effect on the muscular force (F(4, 16) = 3.30, p = 0.211 × 10^−4^) and the range of motion (F(3, 6) = 12.09, p = 0.558 × 10^−10^).

**Figure 3:**
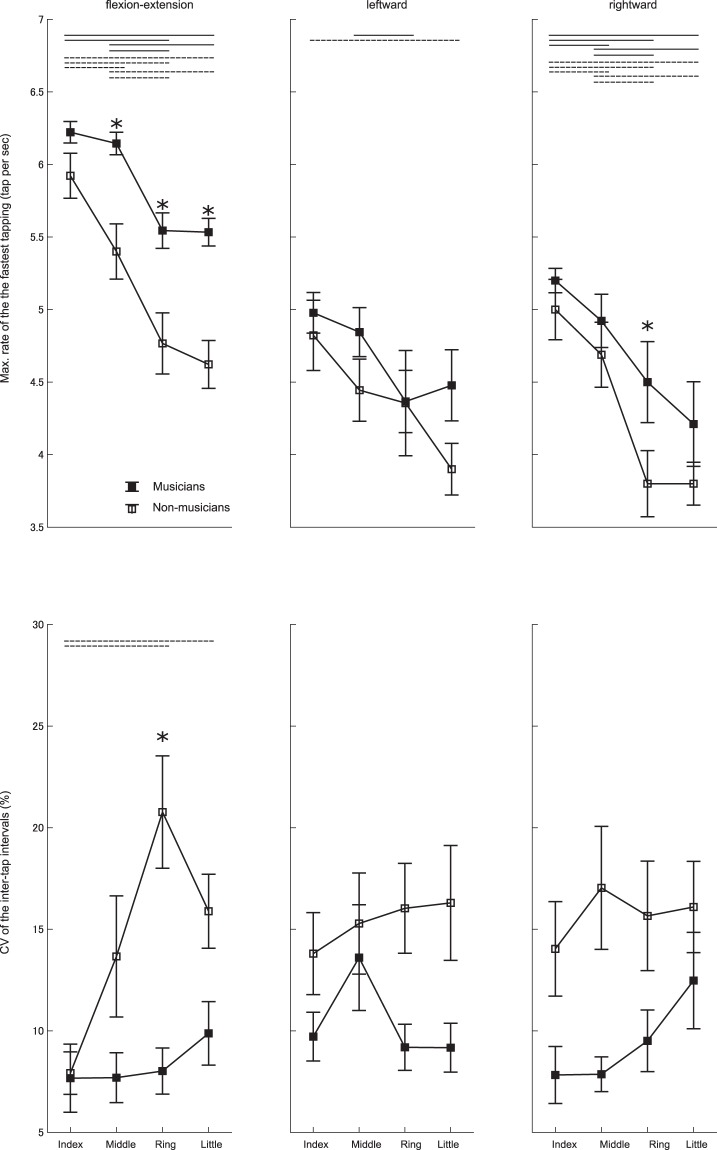
Group means of the maximum finger tapping rate (upper panel) and coefficient of variation (CV) of the inter-tap interval during the fastest tapping (lower panel) with each of the index, middle, ring, and little fingers in the flexion-extension (right panel), leftward (middle panel), and rightward (left panel) directions in the pianists (filled square) and non-musicians (open square). A solid and dotted horizontal line indicates a significant difference between the fingers and between the pianists and non-musicians, respectively (p < 0.05). *ignificant group difference (p < 0.05). An error bar indicates one SEM.

Figure 3 displays the group means of the maximum finger tapping rate and CV of the inter-tap intervals for each of the fingers in the pianists and non-musicians. For the maximum finger tapping rate, MANOVA yielded a significant group effect (F(1, 3) = 15.75, p = 6.321 × 10^−8^), but neither group and finger interaction nor finger effects was significant. Two-way mixed-design ANOVAs also demonstrated significant effects of group for both the flexion-extension (F(1, 3) = 44.63, p = 4.269 × 10^−9^) and the rightward motion (F(1, 3) = 6.43, p = 0.013) but not for the leftward motion (F(1, 3) = 3.10, p = 0.082). Post-hoc tests identified group differences at the middle, ring, and little fingers for the flexion-extension, and at the ring finger for the rightward motion. For the CV of the inter-tap intervals, MANOVA yielded a significant group effect (F(1, 3) = 9.87, p = 1.65 × 10^−5^) but neither interaction effect of group and finger nor main effect of finger was significant. Two-way mixed-design ANOVAs found a significant interaction effect only for the flexion-extension direction (F(3, 9) = 3.61, p = 0.017), and also a significant group effect for each of the flexion-extension (F(1, 3) = 21.52, p = 1.53 × 10^−5^), rightward (F(1, 3) = 11.42, p = 0.001), and leftward motions (F(1, 3) = 16.86, p = 1.05 × 10^−4^). Post-hoc tests found a significant group difference only at the ring finger during the flexion-extension tapping. These findings confirmed that faster tapping in the pianists was not attributed to a tradeoff between speed and accuracy. We therefore used the maximum finger tapping rate in the regression model of study 2 as a measure of the finger dexterity.

### Study 2: Individual differences in the maximum finger tapping rate between expert pianists

**Figure 4:**
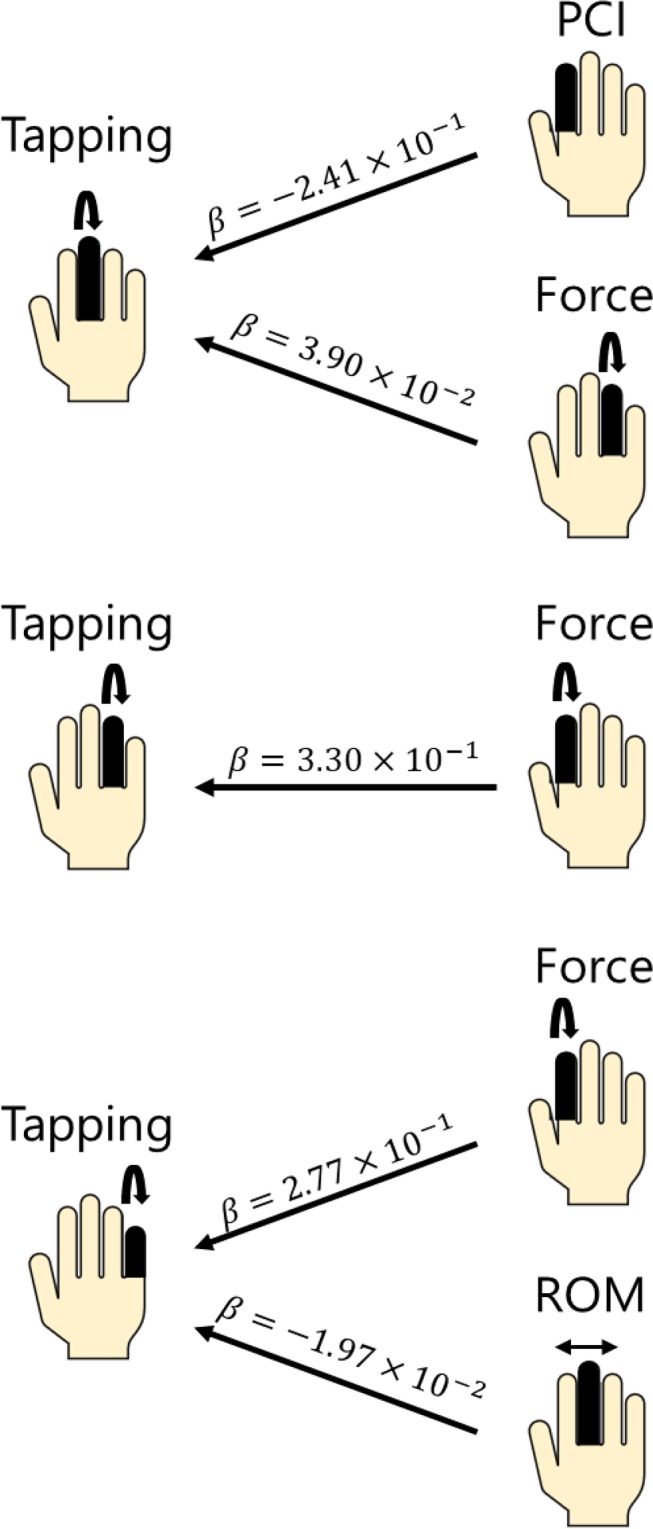
Visualized results of the elastic net regression (Table 2). Variables accounting for the maximum finger tapping rate with each of the middle, ring, and little finger are displayed at the right column. The beta value indicates the coefficient derived from the regression.

Table 2 and Fig. 4 summarizes the results of the elastic net regression for the maximum finger tapping rate in the pianists who participated in study 2. The regression selected a few finger functions associated with the maximum tapping rate of each finger. Here, the regression model was developed only for the maximum tapping rate of the fingers and directions with a significant group difference yielded by study 1. For the flexion-extension tapping, the maximum tapping rate of the middle finger was associated negatively with the PCI of the index finger and positively with the maximum flexion force of the ring finger. The maximum tapping rate of the ring finger covaried positively with the maximum flexion force of the index finger. The maximum tapping rate of the little finger was associated positively with the maximum flexion force of the index finger and negatively with the range of motion of the middle finger at the passive condition. For the rightward tapping with the ring finger, the regression demonstrated that none of the variables representing neuromuscular and biomechanical constraints of the fingers and the finger muscular strength covaried with the maximum tapping rate. Importantly, the elastic net demonstrated that none of the maximum finger tapping rates covaried with age of starting piano practicing and the total amount of piano practicing. In summary, these findings indicate that the maximum finger tapping rate of the expert pianists was associated with the biomechanical features of the hand (i.e. passive range of motion and PCI) and maximum finger muscular force, both of which displayed no significant group difference in study 1. In addition, none of these predictors had a significant correlation with age of starting piano practicing and the total amount of piano practicing (p > 0.05).

**Table 2:** Results of elastic net multiple regression analyses for the fast individual finger movements of pianists.

Dependent variables	max. tap rate 3	max. tap rate 4	max. tap rate 5
Independent variables	PCI2 −2.41 × 10^−1^	MVC Flexion2 3.30 × 10^−1^	MVC Flexion2 2.77 × 10^−1^
	MVC Flexion4 3.90 × 10^−2^		Passive ROM3 −1.97 × 10^−2^
Alpha	0.99	0.99	0.99
Lambda	0.33	0.35	0.36

MVC: maximum voluntary contraction.

ROM: range of motion.

2, 3, 4, 5 in the variables indicates the index, middle, ring, and little finger, respectively.

Since the elastic net can select an independent variable with the strongest covariation with a dependent variable when independent variables covary with together^42^, we further ran a correlation analysis between the independent variables in order to understand the results of study 2 accurately. The PCI of the index finger had no significant correlation with that of each of the other fingers (p > 0.05). By contrast, the muscular strength for the ring finger flexion was strongly correlated with that for flexion of each of the index (r = 0.83, p = 2.52 × 10^−4^), middle (r = 0.86, p = 6.95 × 10^−5^), and little fingers (r = 0.87, p = 6.44 × 10^−5^). Likewise, the muscular strength for the index finger flexion was correlated with that for flexion of each of the middle (r = 0.94, p = 6.46 × 10^−7^), ring (r = 0.83, p = 2.52 × 10^−4^), and little fingers (r = 0.80, p = 6.60 × 10^−4^). The strong correlation of the muscular strength between the fingers indicates that the maximum tapping rate covaried not with the strength of a specific finger, but with that of multiple fingers. Finally, the range of motion of the middle finger was correlated only with that of the ring finger (r = 0.81, p = 2.87 × 10^−4^).

## Discussion

The present results consist of two major observations. First, compared with non-musicians, pianists exhibited faster and more individuated movements of the fingers. Faster finger tapping for the pianists than the non-musicians confirmed that this measure represented a feature of the finger dexterity of the trained individuals. Particularly, the increased tapping rates for flexion-extension at the middle, ring, and little fingers and for the rightward motion at the ring finger in the pianists are likely to be endowed by piano training. In addition to smaller active contribution index (ACI) in the pianists, a lack of any group differences in passive contribution index (PCI), the range of motion, and the muscular strength provided evidences of reduced neuromuscular but not biomechanical constraints of the fingers through piano practicing. Second, an individual difference in the maximum finger tapping rate between the trained pianists covaried with the range of motion, PCI, and muscular strength of the fingers, but not with ACI. The results indicate that both the muscular strength and a measure of mechanical coupling between the fingers, which had no group difference, account for the individual difference in the finger dexterity measure across pianists. Importantly, none of these finger characteristics was correlated with early and deliberate piano practicing, which includes age of starting to play the piano and total duration of piano practicing. Importantly, the contrasting observations between these two results highlight distinct differences in biological factors associated with the effects of piano practicing on and between-pianists differences in the finger motor dexterity. Piano practice is likely to reduce neuromuscular constraints of the fingers to some extent so as to improve the independent movement control, but not necessarily ensure mastery of the finger motor virtuosity of the experts due to the biomechanical constraints and limited muscular strength.

The superior independence of movements between the fingers in pianists to that of non-musicians accompanied reduced neuromuscular constraints of the fingers without differences in biomechanical constraints. This finding extends the previous behavioral studies that demonstrated enhanced movement individuation between the fingers through musical training^18,43^. Some neurophysiological adaptation can be proposed as a putative mechanism underlying the reduced neuromuscular constraints, although the present study did not directly evaluate neurophysiological functions responsible for the finger movements. Using transcranial magnetic stimulation, several studies demonstrated neuroplastic adaptation of the motor cortical functions responsible for dexterous finger movements through musical training^9,26,30,44^. For example, patterns of movement coordination between the fingers, which are encoded in the motor cortex, differed between the pianists and non-musicians^9^. Motor cortical adaptation caused by musical training also includes reduced surround inhibition in musicians, which strengthens coupling between multiple finger muscles^26^. This suggests that the enhanced movement individuation between the fingers result not from enhanced functional individuation between the adjacent finger muscles, but from neuroplastic alteration of finger muscular coordination so that force production at a finger muscle accompanies the production of adjacent-fingers’ muscular force counteracting against neuromuscular and mechanical coupling between the fingers.

A key finding of our study was the association of the individual differences in the finger motor dexterity between the pianists with both mechanical coupling between the fingers and muscular force strength but not with early and deliberate musical training nor with neuromuscular constraints on the finger motor independence. A lack of any relationship of the finger dexterity of the pianists with deliberate musical practice corroborates a concept of gene-environment interaction in acquisition of motor virtuosity^37,38,45^. Possible hereditary factors include innate individual differences in physiological properties of muscles^46,47^ and hand anatomy^48^. In addition, the way of practicing can also play a role in mastering the finger motor skills^36^. For example, the effects of piano practicing on the finger movement independence depend on explicit attention to movement accuracy^43^. The acquired movement coordination between the fingers through practicing the piano also differs between implicit and explicit motor learning^44^. Furthermore, training and exercise being performed independently of musical practicing, such as muscle strength training and stretching to open the hand, may also aid in sophisticating finger motor skills, on the basis of our finding that the finger muscular strength and inter-finger mechanical coupling covaried positively with the maximum speed of the finger movements, although our results are correlational and any causal relationships are not yet demonstrated. Although a causal relationship of these finger characteristics with heredity, ways of musical practicing, and training and exercising independent of musical practicing remains unknown, the present findings can help in optimizing training and practicing of acquiring and sophisticating finger motor skills. Due to a large number of potential biological factors associated with the finger dexterity, it is difficult to specify a target for motor training and rehabilitation. This makes it difficult for experts to master motor skills even through long-term training, which often leads to overtraining and thereby triggers maladaptive changes in the sensorimotor system such as musicians’ dystonia^11^. Our findings and methodologies successfully identified a small number of factors associated with the finger dexterity, which will potentially enable us to focus on a target for motor training and musical practicing for optimizing the finger motor dexterity while reducing risks of overuse syndromes.

A close inspection of the regression results deepens the understanding of mechanisms underlying fast performance of a single finger motion. First, the negative covariation between the middle-finger tapping rate and PCI of the index finger indicates that pianists who can move the middle finger faster had weaker biomechanical connection between the index finger and the other fingers. This result is reasonable due to a lower mechanical constraint of a moving finger with its adjacent finger. For the maximum tapping rate of each of the middle, ring, and little fingers, the muscular strength of a finger covaried positively. However, there were strong correlations between the fingers in terms of the muscular strength. The result thus suggests that pianists with stronger finger muscular strength can perform the finger tapping faster. Finally, a negative covariation of the maximum tapping rate of the little finger with the range of motion of the middle finger indicates that pianists who can move the little finger faster had smaller range of motion at the middle finger. This is puzzling and hard to be interpreted, and therefore future studies should investigate causal effects of stretching of the finger on the maximum tapping rate in order to better understand the underlying mechanism.

There are some limitations of the present study. First, as a measure of manual dexterity, the present study used the maximum finger tapping rate. Although we also assessed accuracy of the finger tapping, this measure represents one of various skills forming the dexterity. Second, one may wonder if the fastest tapping with each of the fingers reflects pianistic skills, since it differs from sequential finger movements during playing a musical excerpt. However, in performance of such tasks, not only motor functions (e.g. independent control of movements between fingers, muscular strength) but also both sensory (e.g. feedback control based on auditory and proprioceptive afferent information^34,49,50^) and cognitive (e.g. chunking, co-articulation^51,52^) functions play key roles, which are not scopes of the present study but our previous studies^34,49,50^. A significant difference in the maximum finger tapping rate between the pianists and non-musicians confirmed that this skill represents a part of pianistic skills, but does not cover their whole aspects. This may underlie a lack of correlation of the present dexterity measure with both early piano practicing and the amount of piano practicing. However, there is another possibility that no correlation between the dexterity and training quantity is due to that transfer effects from piano practice to isolated finger movements occurred during early stages of piano training and stagnated during further development due to physiological limitations.

## Data Availability

The datasets generated during and/or analyzed during the current study are available from the corresponding author on reasonable request.
